# POD-1/*TCF21* Reduces SHP Expression, Affecting *LRH-1* Regulation and Cell Cycle Balance in Adrenocortical and Hepatocarcinoma Tumor Cells

**DOI:** 10.1155/2015/841784

**Published:** 2015-09-02

**Authors:** Monica Malheiros França, Bruno Ferraz-de-Souza, Antonio Marcondes Lerario, Maria Candida Barisson Villares Fragoso, Claudimara Ferini Pacicco Lotfi

**Affiliations:** ^1^Department of Anatomy, Institute of Biomedical Sciences, University of São Paulo, 05508-000 São Paulo, SP, Brazil; ^2^Laboratory of Medical Investigation 18 (LIM-18), School of Medicine, University of São Paulo, 01246-903 São Paulo, SP, Brazil; ^3^Laboratory of Hormones and Molecular Genetics (LIM-42), Adrenal Unit, Division of Endocrinology, School of Medicine, University of São Paulo, 01246-903 São Paulo, SP, Brazil

## Abstract

POD-1/*TCF21* may play a crucial role in adrenal and gonadal homeostasis and represses *Sf-1*/SF-1 expression in adrenocortical tumor cells. SF-1 and LRH-1 are members of the Fzt-F1 subfamily of nuclear receptors. LRH-1 is involved in several biological processes, and both LRH-1 and its repressor SHP are involved in many types of cancer. In order to assess whether POD-1 can regulate LRH-1 via the same mechanism that regulates SF-1, we analyzed the endogenous mRNA levels of *POD-1*, *SHP*, and *LRH-1* in hepatocarcinoma and adrenocortical tumor cells using qRT-PCR. Hereafter, these tumor cells were transiently transfected with pCMVMycPod-1, and the effect of POD-1 overexpression on E-box elements in the *LRH-1* and *SHP* promoter region were analyzed by ChIP assay. Also, Cyclin E1 protein expression was analyzed to detect cell cycle progression. We found that POD-1 overexpression significantly decreased *SHP*/SHP mRNA and protein levels through POD-1 binding to the E-box sequence in the *SHP* promoter. Decreased *SHP* expression affected *LRH-1* regulation and increased Cyclin E1. These findings show that POD-1/*TCF21* regulates SF-1 and LRH-1 by distinct mechanisms, contributing to the understanding of POD-1 involvement and its mechanisms of action in adrenal and liver tumorigenesis, which could lead to the discovery of relevant biomarkers.

## 1. Introduction

POD-1 (Capsulin, Epicardin, and* Tcf21*) is a basic helix-loop-helix (bHLH) transcriptional regulatory protein expressed in mesenchyme cells at sites of epithelial-mesenchyme interactions in the developing urogenital, cardiovascular, respiratory, and gastrointestinal systems [1–4]. In the adrenal gland, POD-1 is expressed exclusively in the capsule region of the adrenal cortex, as shown in mice expressing a lacZ gene reporter under control of the regulatory region of POD-1 [5]. Recently, a progenitor population in the adrenal gland, characterized by the expression of WT1, GATA4, GLI1, and TCF21, was identified by cell lineage tracing analyses and used to generate steroidogenic cells* in vivo* [6]. In the testes of fetal mice, POD-1 repressed Steroidogenic Factor-1 (*Sf-1*/*Nr5a1*), a transcription factor required for gonadal development and sex determination, and regulated adrenal and gonadal steroidogenesis in adult mice [7, 8]. In human adrenocortical tumor cells, we found that POD-1 binds to the* SF-1* E-box promoter sequence and inhibits SF-1 expression, as well as the expression of StAR protein, which is controlled by SF-1 [9].

The orphan nuclear receptor Liver Receptor Homologue-1 (*LRH-1*/*NR5A2*) also belongs to the Fzt-F1 subfamily of nuclear receptors [10]. LRH-1 is involved in development, steroidogenesis, and proliferation of liver, pancreas, and intestine cells [11, 12]. LRH-1 is critical for liver development and regulates cholesterol and bile acid homeostasis in the adult liver [11, 12]. The human adrenal gland also expresses LRH-1, although LRH-1 expression has been described to be species-specific [13, 14]. LRH-1 is active constitutively and its transcriptional activity is repressed by orphan receptors like the Small Heterodimer Partner (SHP/*NR0B2*) and the dosage-sensitive sex reversal-adrenal hypoplasia congenital gene on the X chromosome (DAX-1) [15, 16]. Like DAX-1, an important nuclear receptor involved in adrenal development, SHP is an unusual orphan nuclear receptor because it lacks a DNA-binding domain [17]. SHP interacts with the AF-2 transactivation domain of LRH-1 and competes with the binding of coactivators [18]. In primary hepatocarcinoma cells and nude mice, SHP/*Shp* inhibited tumor growth and induced apoptosis [19]. SHP also inhibits estrogen action at multiple levels, and its induction of expression or activity in the breasts is relatively specific for inhibiting estrogen signaling in breast cancer [20]. LRH-1 is also involved in tumorigenesis in pancreatic and intestinal cancer [21, 22]. Its suppression arrested the cell cycle, mediated by the downregulation of Cyclin E1, in human hepatocellular carcinoma cells [23]. Despite our previous work demonstrating that POD-1 overexpression binds and inhibits SF-1 expression, its mechanism of action in the other members of the Fzt-F1 nuclear receptor family, like LRH-1, is unknown. To fill this gap and explore the effect of POD-1 in cell cycle regulation in tumor cells, we investigated whether POD-1/TCF21 regulates LRH-1 and SHP in adrenocortical and hepatocarcinoma cell lines. In this study, we show that POD-1/*TCF21* reduces* SHP* expression in hepatocarcinoma cells, resulting in an increased* LRH-1* and Cyclin E1 expression via binding in the E-box element of* SHP*, through a different mechanism of action and effect other than that described for SF-1.

## 2. Materials and Methods

### 2.1. Cell Cultures

The NCI-H295R human adrenocortical tumor cell line [24] was cultured in RPMI (Gibco, USA), supplemented with 2% fetal bovine serum (Gibco, USA) and 1% ITS (Gibco, USA). The HepG2 human hepatocarcinoma tumor cell line [25] and the ACC-T36 human adrenocortical tumor cell culture [9] were cultured in DMEM (Gibco, USA) and supplemented with 10% fetal bovine serum (Gibco, USA). The Y-1 mouse adrenocortical tumor cell line [26] was grown in DMEM supplemented with 7.5% horse serum (Gibco, USA) and 2.5% fetal bovine serum (Gibco, USA). All cells were maintained at 37°C in a humidified atmosphere of 95% air and 5% CO_2_.

### 2.2. Cell Culture Transfection

The H295R and HepG2 cell lines were transiently transfected with pCMVMycPod-1, which was kindly provided by Dr. Masataka Nakamura (Tokyo Medical University, Japan), as described in Funato and coworkers [27]. To extract RNA, we plated 1.5 × 10^5^ cells into 6-well tissue culture plates (Becton Dickinson Labware, Franklin Lakes, NJ, USA) and transfected them with 2 *μ*g of plasmid DNA combined with 2 *μ*L of X-tremeGENE HP-DNA transfection reagent (Roche Diagnostics GmbH, Mannheim, Germany). For the protein assay, we plated 5 × 10^5^ cells into a 60 mm tissue culture dish (Becton Dickinson Labware, Franklin Lakes, NJ, USA) and transfected them with 5 *μ*g of plasmid DNA and 5 *μ*L of X-tremeGENE HP-DNA transfection reagent per 1 × 10^5^ cells (Roche Diagnostics GmbH, Mannheim, Germany). For the ChIP assay, we plated 6.6 × 10^5^ cells into a 100 mm tissue culture dish and transfected them with 10 *μ*g of plasmid DNA and 10 *μ*L of X-tremeGENE HP-DNA transfection reagent (Roche Diagnostics GmbH, Mannheim, Germany).

### 2.3. Total RNA Extraction and Quantitative Reverse Transcription PCR (qRT-PCR)

After 48 h of transfection, we extracted total RNA using Trizol reagent (Invitrogen, USA) and treated it with TURBO DNA-free (Ambion). The cDNA was generated from 1 *μ*g of treated total RNA using Oligo dT, RNaseOUT, and M-MLV Reverse Transcriptase (Invitrogen, Carlsbad, CA, USA) following the manufacturer's instructions. The primers used for qRT-PCR were* hPOD-1* forward 5′-ACCCTCTTCCTCGCTTTCTC-3′ and reverse 5′-AACCCGTCACATTCCAACAT-3′;* hLRH-1* forward 5′-TGCCTTGCCTCCTACAGACT-3′ and reverse 5′-AGGCTCATCTGGCTCACACT-3′;* mLrh-1* forward 5′-ACCTGTGAGCCCTGAAGCTA-3′ and reverse 5′-AGAGGGTTACTGCCCGTTTT-3′;* hSHP-1* forward 5′-CACTGGGTGCTGTGTGAAGT-3′ and reverse 5′-CCAATGATAGGGCGAAAGAA-3′. RT-qPCR was performed on a RotorGene6000 Corbett (Qiagen, USA) sequence detector using Platinum SYBR qPCR SuperMix-UDG (Invitrogen, USA). A cycle threshold (Ct) value in log range of amplification was selected for each sample in triplicate and was normalized to *β*-actin expression levels. Reactions were carried out in triplicate. A pool of commercially available RNA isolated from normal adrenal gland of 62 male/female Caucasians, aged 15–61, was used as a normal control (Clontech, Palo Alto, CA). Data were analyzed using the 2^−ΔΔCt^ method [28].

### 2.4. Immunoblotting

The cells were lysed 72 h after transfection in RIPA buffer, as well as in protease and phosphatase inhibitors (Sigma Aldrich Gmbh, Steinheim, Germany). Bradford assay determined the total protein concentration. Total protein from the lysates (30 *μ*g) was resolved by 12% SDS-PAGE and, after electrophoresis, gels were blotted onto nitrocellulose membranes. Nonspecific binding sites were blocked for 2 h with 0.1% bovine serum albumin or 5% nonfat dried milk in TBST (TRIS-buffered saline solution containing 1% Tween 20). All washes and antibody incubations were performed using TBST. The following primary antibodies were used: anti-Cyclin E1 (Sta Cruz sc-481) 1 : 500 in blocking buffer (0.1% bovine serum albumin in TBST), anti-SHP (Perseus Proteomics-PP-N7519-00) 1 : 1000 in blocking buffer (5% nonfat dried milk in TBST), and anti-actinin 1 : 1000 in TRIS-buffered saline containing 1% Tween 20. Proteins were visualized by ECL detection with secondary HRP-conjugated anti-rabbit (Amersham Hybond ECL, Freiburg, Germany) or anti-mouse antibodies (Jackson Immuno Research, Pennsylvania, USA). Immunoblot results were quantified by densitometer using the GeneSnap and GeneTools software (SynGene-Synoptic Ltd., Cambridge, UK). Ponceau staining of membranes was used to monitor protein transfer and loading.

### 2.5. Chromatin Immunoprecipitation (ChIP)

HepG2 cells transfected with pCMVMycPod-1 were fixed with 1% formaldehyde for 10 min. ChIP assays were performed using the ChIP-IT Express kit (Active Motif, Rixensart, Belgium) following the manufacturer's instructions. Chromatin was fragmented by sonication with eight 10-s pulses at 25 *μ*m amplitude in a VCX130PB ultrasonic processor (Sonics & Materials, CT, USA). Most resulting chromatin fragments ranged from 200 bp to 600 bp. Sheared chromatin was incubated with 3 *μ*g anti-MYC (Clontech) or with 1 *μ*g IgG as negative control (ChIP-IT Control Kit-Human, Active Motif).

### 2.6. ChIP-PCR

The 5.0-kb upstream sequences of human Liver Receptor Homolog-1 (*NR5A2*,* transcript *ENST00000367362,* Ensembl release 64*,* GRCh37*), human Small Heterodimer Partner (*NR0B2*-001 ENST00000254227,* Ensembl release 64*,* GRCh37*), and androgen receptor (*AR, transcript ENST00000374690, Ensembl release 64*,* GRCh37*) were analyzed for putative E-box binding sites using MatInspector [29]. Based on this analysis and on the location of predicted E-box sites in humans [9], the following primers were designed using Primer3 [30]:* AR* E-box, forward 5′-CTCTGATTCTTGGGGCTGAG-3′ and reverse 5′CATGACCAAGCCAGCAGATA 3′ (113 bp amplicon);* LRH-1* E-box-53, forward 5′-TCATTTCTTTGCCATTATCTGG-3′ and reverse 5′-TGGAAACTTTTGATAGGCTTTGA-3′ (120 bp amplicon);* LRH-1* E-box-1300, forward 5′-CCCATACACACAACCTGCAT-3′ and reverse 5′-TGCTGGAATTATAGGCGTGA-3′ (100 bp amplicon);* SHP* E-box-117, forward 5′-ACCGGCCACTTCATTGACT-3′ and reverse 5′-CCAACAACCTTGACTCCAGAA-3′ (146 bp amplicon);* SHP* E-box-3702, forward 5′- CAGGTATGCACCACCATGTC-3′ and reverse 5′-ATCTCAGCACTTTGGGAAGG-3′, (131 bp amplicon).

As a negative control for POD-1 binding, primers amplifying sequences in intron 1-2 of* AR* (forward 5′-TTGTCAAAGTCTTTTCCAGTTAATTT-3′ and reverse 5′-TTAACCCTACCAAGTAAATTTGTTC-3′, 114 bp amplicon), in intron 2-3 of* LRH-1* (forward 5′-CCCACTGGAAGGTGATCCTA-3′ and reverse 5′-CCCCTTTGTCTTTCCCCTTA-3′, 97 bp amplicon), and in intron 1-2 of* SHP* (forward 5′-GGGAGGACAGGAAAGGAGTC-3′ and reverse 5′-CCTGGGGAACTCTCATCTCA-3′, 104 bp amplicon) were used. Anti-MYC-IP, IgG-IP, and 0.1% input DNA samples were used as templates for PCR amplification. PCR reactions were performed using 5 U/*μ*L Platinum Taq DNA Polymerase (Invitrogen) and 5 *μ*M primers for 35 cycles of amplification with an annealing temperature of 60°C.

### 2.7. Statistical Analyses

Data are presented as mean ± standard deviation (SD) values of three replicate experiments. Data were analyzed using the Kruskal-Wallis test (nonparametric one-way ANOVA) or with paired *t*-tests, when indicated. Results were considered statistically significant when *p* < 0.05.

## 3. Results

### 3.1. Distinct Endogenous* POD-1*,* SHP*, and* LRH-1* mRNA Levels Found in Tumor Cells

**Figure 1:**
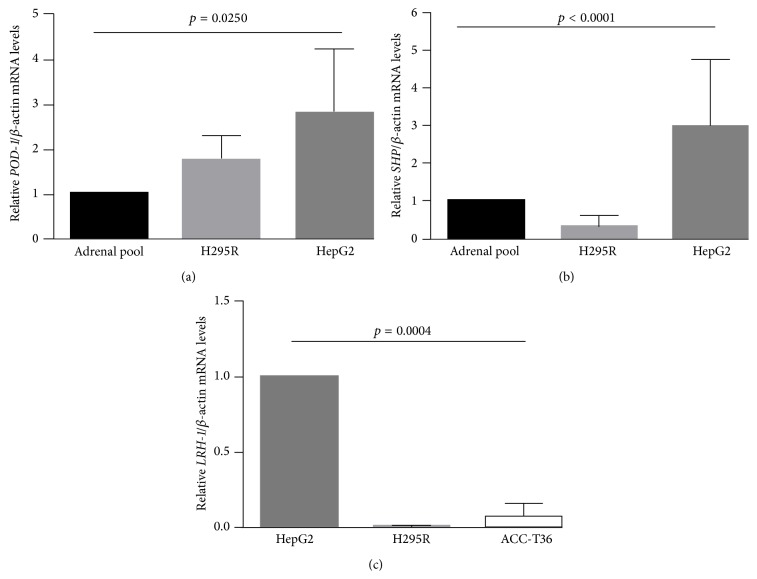
Quantitative reverse transcription PCR (qRT-PCR) analysis of the relative* POD-1*/*β-actin* (a),* SHP*/*β-actin* (b), and* LRH-1*/*β-actin* mRNA levels (c) in the human adrenocortical tumor cell line (H295R and ACC-T36 cells) and in the human hepatocarcinoma tumor cell line (HepG2 cells). Differences were tested with a Kruskal-Wallis one-way ANOVA. Values represent means ± standard deviations from 3 experiments.

We used qRT-PCR to estimate the endogenous mRNA levels of* POD-1*,* SHP*, and* LRH-1* in HepG2 and H295R tumor cell lines and in a human tumor adrenocortical cell culture, ACC-T36 cells, using qRT-PCR (Figure 1).* POD-1* was significantly higher in H295R and HepG2 cells (by 1.73 ± 0.27- and 2.8 ± 0.79-fold, resp.) than in the normal adrenal pool (*p* = 0.025; Figure 1(a)). Endogenous* SHP* in both cell lines differed significantly (*p* < 0.0001) from the normal adrenal pool, and* SHP* was higher in HepG2 than H295R, respectively, by 2.99 ± 1.8- and 0.3 ± 0.2-fold (Figure 1(b)). In contrast,* LRH-1* was barely detectable in H295R cells (0.006 ± 0.002-fold, *p* = 0.0004) and ACC-T36 cells (0.07 ± 0.03-fold, *p* = 0.0004) when compared to HepG2 cells (Figure 1(c)).

### 3.2. POD-1 Overexpression Reduces SHP Expression in HepG2 and H295R Cells

**Figure 2:**
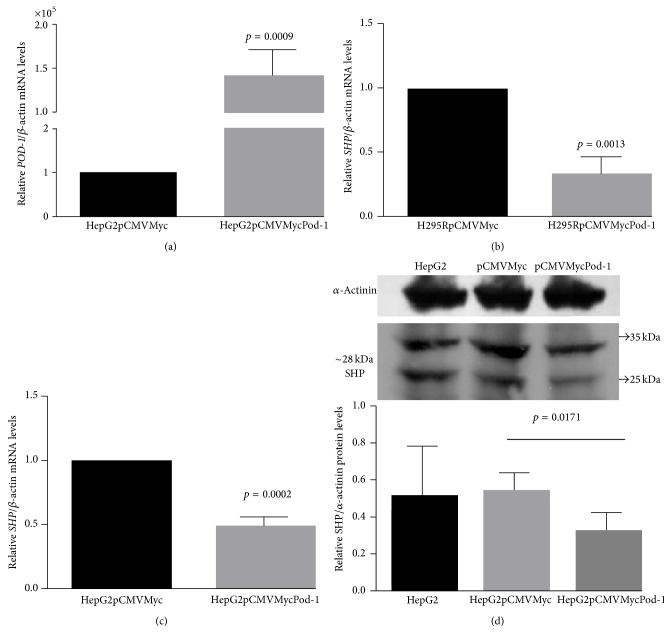
Quantitative reverse transcription RT-PCR (qRT-PCR) and immunoblotting analysis of relative* SHP*/SHP expression. Relative* POD-1*/*β-Actin* mRNA levels in HepG2 cells transiently transfected with the empty vector pCMVMyc or with pCMVMycPod-1 (a);* SHP*/*β-Actin* mRNA levels in H295R cells transiently transfected with the empty vector pCMVMyc or with pCMVMycPod-1 (b);* SHP*/*β-Actin* mRNA levels and SHP/*α*-actinin protein levels in HepG2 cells (c and d, resp.). Total RNA samples and protein samples were prepared 48 h and 72 h after transfection, respectively. Differences were tested with paired samples *t*-tests. Values represent means ± standard deviations from 3 experiments.

The transient transfection of pCMVMycPod-1 in HepG2 cells increased* POD-1* mRNA levels (143.469 ± 16072-fold, *p* = 0.0009) compared to controls transfected with the empty vector (pCMVMyc) (Figure 2(a)). The* POD-1* mRNA in H295R and ACC-T36 cells transfected with pCDNA3Pod or pCMVMycPod-1 showed an increase, as previously shown in França et al. [9]. Then, we determined the effect of overexpression of* POD-1* on* SHP*/SHP mRNA and protein levels in H295R and HepG2 cells transiently transfected with the expression vector pCMVMycPod-1 (Figure 2(d)).* SHP* mRNA levels of H295R and HepG2 cell lines were reduced by 3.7 ± 0.08-fold (*p* = 0.0013; Figure 2(b)) and 2.3 ± 0.04 (*p* = 0.0002; Figure 2(c)), respectively, compared to cells transfected with the empty vector.* SHP* protein levels, showing two putative variants with approximately 28 kDa, were also significantly lower in HepG2 cells (0.29 ± 0.04 fold) than in controls (*p* = 0.017; Figure 2(d)).

### 3.3. Validation of Chromatin Enrichment and Characterization of* SHP*-E-box Binding of POD-1

**Figure 3:**
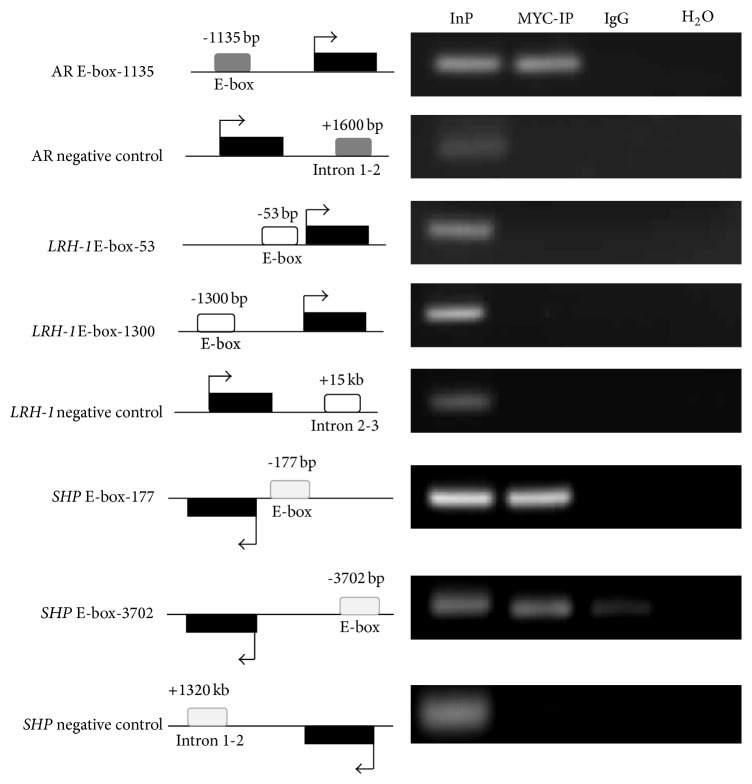
Chromatin enrichment was confirmed by PCR amplification of the E-box region of the* SHP-1* promoter from anti-MYC immunoprecipitated HepG2pCMVMycPod-1 DNA. The positions of the amplicons in relation to the Transcriptional Start Sites (TSSs) (represented by arrows) are shown. Black and open bars represent the exon and a different E-box sequence, respectively. Androgen receptor (*AR*), input (Inp) 0.1% DNA, Anti-MYC-IP HepG2pCMVMycPod-1 (IP), and anti-IgG (IgG).

To determine whether the repressive effect of POD-1 overexpression on SHP expression was mediated through POD-1 binding to E-box elements in the SHP promoter region, chromatin immunoprecipitation (ChIP) assays were performed in HepG2 cells transfected with pCMVMycPod-1 using an anti-Myc antibody (Figure 3). To validate this approach, ChIP-PCR assays were performed using primers amplifying an E-box sequence in the promoter region of* AR* (E-box sequences are compared and shown in Table 1); it has been previously shown in mice and humans that POD-1 binds to an E-box element in the promoter of* Ar/AR* to repress transcription [9, 31]. As shown in Figure 3, immunoprecipitated chromatin was enriched for this region, whereas intron 1-2 of* AR*, used as a local negative control and not expected to bear E-box elements, failed to amplify. The 5.0-kb upstream sequence of human* SHP* and* LRH-1* was analyzed for putative E-box elements using MatInspector [29], and two* SHP* elements were identified, located 177 and 3702 base pairs upstream of the transcription start site, respectively. Two* LRH-1* elements were also identified, located 53 and 1300 bases pairs upstream of the transcription start site. ChIP-PCR assays using specifically designed primers showed that Myc immunoprecipitated DNA from HepG2-pCMVMycPod-1 cells amplified both “E-box-177” and “E-box-3702” sequences. This confirms that chromatin was enriched by ChIP, while the negative control sequence located in the intron 1-2 (1320 Kb) of* SHP* failed to amplify (Figure 3). Nevertheless, as shown in Figure 3, MYC-IP DNA from HepG2 transfected cells did not amplify “E-box-53” and “E-box-1300”* LRH-1* sequences, as well as the ChIP negative control sequence (intron 2-3). Altogether, these results show POD-1 binding to E-box elements in the human* SHP* promoter site.

### 3.4. The Downregulation of SHP Induced by POD-1 Increased* LRH-1* mRNA and Cyclin E1 Protein Levels in HepG2 Cells

**Figure 4:**
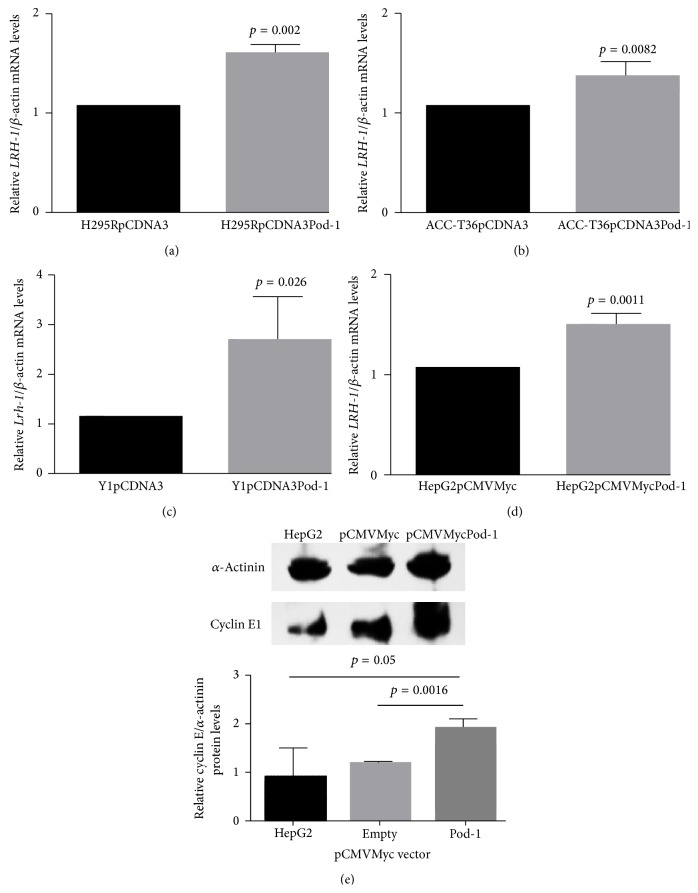
Quantitative reverse transcription RT-PCR (qRT-PCR) of* Lrh-1*/*LRH-1* mRNA levels and immunoblotting of Cyclin E1 protein levels.* LRH-1*/*β-actin* mRNA levels in H295R cells (a), ACC-T36 cells (b), Y1 cells (c), and HepG2 cells (d) transiently transfected with the empty vector (pCDNA3 or pCMVMyc) or with pCDNA3Pod-1 or pCMVMycPod-1; immunoblotting analysis of the relative Cyclin E1/*α*-Actinin protein levels in HepG2 cells (e) transiently transfected with the empty vector pCMVMyc or with pCMVMycPod-1. Total RNA samples and protein samples were prepared 48 h and 72 h after transfection, respectively. Differences were tested with paired samples *t*-tests. Values represent means ± standard deviations of 3 experiments.

To evaluate the effect of POD-1-mediated inhibition of* SHP*/SHP in adrenocortical carcinoma and hepatocarcinoma cells transfected with POD-1,* LRH-1*/*Lrh-1* mRNA and Cyclin E1 protein levels were analyzed by qRT-PCR and immunoblotting (Figure 4).* LRH-1* mRNA levels were significantly increased in human and mice adrenocortical tumor cells relative to controls (H295R cell line: 1.61 ± 0.04-fold, *p* = 0.002, Figure 4(a); ACC-T36 cells: 1.38 ± 0.07-fold, *p* = 0.0082, Figure 4(b); and Y1 cell line: 2.71 ± 0.4-fold, *p* = 0.026, Figure 4(c)).* LRH-1* mRNA levels were also increased in HepG2 cells transfected with pCMVMycPod-1, compared to transfected controls (1.51 ± 0.06-fold, *p* = 0.0011, Figure 4(d)). POD-1 overexpression also significantly increased Cyclin E1 protein levels in the HepG2 cell line relative to controls transfected with pCMVMyc (1.94 ± 0.09 fold, *p* = 0.0016). Taken together, these results could be associated with a decrease of SHP-mediated protein expression.

## 4. Discussion

Here we described that SHP is regulated by POD-1/*TCF21* through binding E-box sequences to the SHP promoter in adrenocortical and hepatocarcinoma tumor cells. SHP repression affected* LRH-1* regulation and cell cycle balance, which was associated with increased Cyclin E1 levels.

Previous studies show that POD-1 may play a crucial role in adrenal and gonadal homeostasis, and it has also been described to play a role in different organs [1, 3, 8]. Furthermore,* TCF21* is downregulated in adrenocortical carcinoma (ACC), melanoma, lung, and head and neck squamous cell carcinomas [9, 32–34]. Consistent with these findings, our results showed that* POD-1*,* SHP*, and* LRH-1* are expressed in adrenocortical and hepatocarcinoma tumor cells.

POD-1 has been shown to repress* Sf-1*/SF-1 expression in mouse and human adrenocortical cells [8, 9], in contrast with what was observed here with LRH-1, which is upregulated by inhibition of SHP. SF-1 and LRH-1 have structural similarities; however, they also have important functional differences [10]. LRH-1 and SHP are involved in many types of cancer such as liver, pancreatic, gut, and breast [17]. SHP appears to suppress tumorigenesis in liver cancer, inhibiting tumor growth and increasing sensitivity to apoptotic stimuli, due to repression of LRH-1-dependent Cyclin D1 transcription [35, 36]. In this study, we showed that POD-1 is able to modulate SHP expression in human adrenocortical and hepatocarcinoma cell lines, which provides new insights into the role of POD-1 in both types of cancer. Using a ChIP assay, we showed that POD-1 binds directly to the E-box* SHP* promoter, inhibiting its activity, but does not bind to the* LRH-1* promoter E-box sequence.

In intestinal crypt cells, LRH-1 controls cell proliferation by inducing the expression of G1 Cyclins (D1 and E1) and is correlated with the rate of intestinal cell renewal. The increase of proliferation in these cells occurs after induction of Cyclins D1 and E1, enhanced by the interaction with *β*-catenin [21].* In vivo* and* in vitro* approaches have shown that LRH-1 overexpression promoted pancreatic cancer cell growth, proliferation, and angiogenesis by regulating Cyclins E1 and D1 [37]. Furthermore, LRH-1 has been shown to contribute to colon tumorigenesis* in vivo* through its effects on the cell cycle and inflammation [38]. LRH-1 is also involved in the progression and development of pancreatic adenocarcinoma cells [22]. Consistent with these findings, we found that SHP downregulation increased LRH-1 expression, which binds to the Cyclin E1 promoter that may lead to the G1 to S phase transition of the cell cycle. These results are in contrast with our finding that POD-1 inhibits SF-1 in tumor adrenocortical cells [9], suggesting that POD-1 has different effects on the control of the cell cycle in tumor cells expressing LRH-1.

## 5. Conclusion

We showed that POD-1 overexpression promotes* LRH-1* increase through downregulation of SHP, which may promote cell cycle progression via Cyclin E. POD-1/*TCF21* has a potential role as a regulator of SF-1 and LRH-1 transcription factors via distinct mechanisms promoting different effects. These findings contribute to the understanding of how the cellular tumorigenic process is controlled in adrenocortical and liver tumors and suggests new therapeutic targets.

## Figures and Tables

**Table 1:** Nucleotide sequence and E-box elements identified *in  silico* using MatInspector.

Identified E-box	Species	Sequence
AR E-box -1135	Human	5′-atgccaCGAGgcc-3′
	Mouse	5′-gt gtcaggaattc-3′

LRH-1 E-box -53	Human	3′-tcatcaCATGact-5′
	Mouse	3′-tggtcacatgacc-5′

LRH-1 E-box -1300	Human	5′-tggccaGGTGcgg-3′
	Mouse	5′-ttgccttcagtgc-3′

SHP E-box -177	Human	5′-gtgccaCGTGggg-3′
	Mouse	5′-aggccacgtggagc-3′

SHP E-box -3702	Human	3′-ggaTCACttgagg-5′
	Mouse	3′-agatctctcagct-3′

E-box elements were named based on distance to transcriptional start, as shown in Figure 3. Core human sequence elements, as identified by MatInspector (see Section 2), are shown in capitals.

Nucleotides conserved, in human and mouse, are shown underlined in the mouse sequence; sequence alignment was done using Clustalw2 (http://www.ebi.ac.uk/Tools/msa/clustalw2/), under default settings.
